# Hepatocellular carcinoma-associated antigen 59 of *Haemonchus contortus* modulates the functions of PBMCs and the differentiation and maturation of monocyte-derived dendritic cells of goats *in vitro*

**DOI:** 10.1186/s13071-019-3375-1

**Published:** 2019-03-14

**Authors:** QiangQiang Wang, LingYan Wu, Muhammad Waqqas Hasan, MingMin Lu, WenJuan Wang, RuoFeng Yan, LiXin Xu, XiaoKai Song, XiangRui Li

**Affiliations:** MOE Joint International Research Laboratory of Animal Health and Food Safety, College of Veterinary Medicine, Nanjing Agricultural University, Nanjing, Jiangsu PR China

**Keywords:** HCA59, *Haemonchus contortus*, Dendritic cells, PBMCs, Immunomodulatory effects

## Abstract

**Background:**

Hepatocellular carcinoma-associated antigen 59 (HCA59), which is one of the most important excretory/secretory products of *Haemonchus contortus* (HcESPs), is known to have antigenic functions. However, its immunomodulatory effects on host cells are poorly understood.

**Methods:**

Here, we cloned the HCA59 gene and expressed the recombinant protein of HCA59 (rHCA59). Binding activities of rHCA59 to goat peripheral blood mononuclear cells (PBMCs) and dendritic cells (DCs) were checked by immunofluorescence assay (IFA) and the immunoregulatory effects of rHCA59 on cytokine secretions, cell migration, cell proliferation, nitric oxide production, and changes in expression of genes in related pathways were observed by co-incubation of rHCA59 with goat PBMCs and DCs. Monocyte phagocytosis and characterization of goat blood DC subsets were detected by flow cytometry.

**Results:**

The IFA results revealed that rHCA59 could bind to PBMCs and DCs. Treatment of PBMCs with rHCA59 significantly increased cellular proliferation and NO production in a dose–dependent manner, while cell migration was vigorously blocked. Treatment with rHCA59 significantly suppressed monocytes phagocytosis. The quantity of surface marker CD80 on DCs increased significantly after rHCA59 treatment. In addition, the expression of genes included in the WNT pathway was related to the differentiation and maturation of DCs, and the production of IL-10 and IL-17 produced by PBMCs was altered.

**Conclusions:**

Our findings illustrated that rHCA59 could enhance host immune responses by regulating the functions of goat PBMCs and DCs, which would benefit our understanding of HCA59 from parasitic nematodes contributing to the mechanism of parasitic immune evasion.

**Electronic supplementary material:**

The online version of this article (10.1186/s13071-019-3375-1) contains supplementary material, which is available to authorized users.

## Background

Haemonchus species are the most important blood-sucking nematodes of domestic animals in sub-tropical and tropical areas worldwide. Infections of these parasites usually appear in young animals and resistance to infection develops with exposure in adult hosts [1]. As blood-feeders, adult *Haemonchus contortus* can lead to severe hemorrhagic gastritis, a low level of proteins in blood, lethargy, edema and lack of red blood cells, and even death in acute infections of the animal [2–4]. Current methods of controlling *H. contortus* primarily depend on the use of anthelmintics. However, extensive resistance to frequently used drugs has led to an urgent need for development of new chemotherapeutics and different control approaches, such as vaccination [5–9].

Hepatocellular carcinoma-associated antigen 59 (HCA59), which is a component of the excretory/secretory products of *H. contortus* (HcESPs), can be isolated from different larval stages of this nematode [10]. In NCBI, HCA59 had 1111 blast hits to 862 proteins in 155 species including archaea, bacteria, metazoa, fungi, plants and viruses. This protein belongs to the TLS1 family, is associated with the spliceosome, and is overexpressed in multiple cancer cell lines in humans [11]. Hepatocellular carcinoma-associated antigen 587 (HCA587) has been found to be the protein most similar to HCA59, and its peptides could bind specifically to DCs [12].

In nematode infections, the parasites regulate the host immune system by affecting the functions of the immune cells, through interactions of ESPs with the cells [13]. PBMCs refer to cells with a single nucleus, including various immune cells, such as lymphocytes (B cells, T cells), monocytes, DCs and NK cells, which play key roles in the host’s immune system. DCs are the only multiple-function cells that can both initiate primary immune responses and manage adaptive and innate immunities through antigen uptake and processing, stimulation of lymphocytes and release of cytokines [14–16]. Earlier studies indicated that DCs maturation processes were predominantly based on the non-canonical and canonical WNTs that stimulate DCs to produce anti-inflammatory cytokines [17]. Therefore, WNT proteins and their signaling pathways play significant roles in DCs function and maturation.

We previously recognized that HCA59 from *H. contortus* interacted with goat PBMCs *in vivo* [10]. However, molecular cloning was not conducted and functional descriptions of *H. contortus* HCA59 were not provided in that study. Therefore, in the present study, recombinant protein of HCA59 was generated and its modulatory effects on the functions of goat PBMCs and the differentiation and maturation of DCs were evaluated.

## Methods

### Animals and parasites

Local crossbred goats (3- to 6-months-old) were housed indoors and provided with whole shelled corn, hay and water *ad libitum*. All goats were dewormed with levamisole (8 mg/kg body weight) to eliminate the naturally attained infection with helminths at 2-week intervals. Fecal samples were analyzed microscopically for helminth eggs twice per week, utilizing standard parasitological techniques. Goats presenting no helminth eggs were used in later experiments.

Female SD rats (body weight ~150 g) were bought from the Experimental Animal Center of Jiangsu, PR China (Certified: SCXK 2008-0004). Rats were housed in a sterilized room and supplied with sterilized water and food.

Third-stage (L3) larvae were obtained from the Laboratory of Veterinary Parasitic Diseases of Nanjing Agricultural University, for use in the challenge and the HcESPs were kept in the same laboratory.

### Cloning and sequence analysis of *H. contortus* HCA59

Total RNA was extracted from adult *H. contortus* parasites (mixture of females and males) isolated from the abomasum of donor goats. RNA was extracted using Trizol reagent (Invitrogen, Shanghai, China) as previously described [18]. Reverse transcription was conducted using a cDNA Kit (Takara Biotechnology, Dalian, China) to synthesize cDNA according to the manufacturer’s instructions, after which the cDNA was stored at -20 °C until use. The open reading frame (ORF) of HCA59 was amplified by PCR using specific primers designed from conserved domain sequences (CDS) of the *H. contortus* HCA59 gene (GenBank: CDJ80864.1). For subsequent cloning, the enzyme restriction site *BamH*I was added to the 5′-end of the sense primer and the enzyme restriction site *Hind*III was added to the 5′-end of the antisense primer. The sequences of sense and antisense primers were as follows: sense: (5′-AAG GAT CCA TGA CGG AAT TCT TCT CGC G-3′) and antisense: (5′-CCA AGC TTC CTC GAG AAG TAT TCG ATG AGC TTC-3′). The total 50 µl PCR reaction consisted of 1.0 U Taq DNA polymerase (Takara Biotechnology), 2 µl of cDNA, 400 µM dNTP mixtures, 3.0 mM MgCl_2_, 50 µM 10× LA PCR buffer (Mg^2+^ free) and 400 nM of each primer. The loop conditions of PCR were as follows: 94 °C for 5 min, followed by 35 cycles of 94 °C (30 s), 55 °C (30 s) and 72 °C (1 min), after which samples were subjected to final extension at 72 °C for 10 min, and preserved at 16 °C until further use. The amplified fragment of PCR was identified by 1% agarose gel electrophoresis, cleansed using an E.Z.N.A. Gel Extraction Kit (Omega Biotech, Norcross, GA, USA), according to manufacturer’s guidelines, then ligated into pMD19-T cloning vector (Takara). The recombinant plasmid pMD-19T/HCA59 was transformed into *E. coli* strain DH5α (Invitrogen Biotechnology, Shanghai, China) and cultured in Luria Bertini medium (LB) containing ampicillin (100 μg/ml). The assured recombinant clones were sequenced (Invitrogen Biotechnology), then validated by sequence analysis utilizing the Blast program online (http://www.blast.ncbi.nlm.nih.gov/blast.cgi).

### Purification of recombinant HCA59 protein

The recognized recombinant plasmid pMD-19T/HCA59 was digested with the double restriction enzymes *BamH*I and *Hind*III, after which the HCA59 gene was purified and ligated into the pET-32a (+) expression vector (Novagen, Shanghai, China) digested with the same enzymes. Finally, to confirm its location in the accurate reading frame, the successfully cloned HCA59 gene in a recombinant expression vector was sequenced. The recombinant plasmid pET-32a (+) with HCA59 was then transferred into *E. coli* BL21 (DE3) and cultured in Luria Bertini (LB) medium containing ampicillin (100 µg/ml). Next, the protein expression was induced with 1 mM isopropyl-β-d-thiogalactopyranoside (IPTG; Sigma-Aldrich, Shanghai, China) until the OD600 of the culture reached 37 °C. The cells were subsequently harvested by centrifugation, after which they were lysed using lysozyme (10 µg/ml) (Sigma-Aldrich) and then sonicated. The sonication products were subsequently examined by 12% (w/v) sodium dodecyl sulfate-polyacrylamide gel electrophoresis (SDS-PAGE). Next, recombinant protein was purified using a Ni^2+^-nitrilotriacetic acid (Ni-NTA) column (GE Healthcare, Piscataway, USA) according to the manufacturer’s methods. The histidine-tagged protein (pET-32a protein) from BL21 transferred with vector used as a negative control protein in this study was also expressed and purified using the same procedures. The purity of the rHCA59 protein was determined by 12% SDS-PAGE with staining by Coomassie blue and quantified by the Bradford method [19]. A ToxinEraserTM Endotoxin Removal Kit (GeneScript, Piscataway, USA) was used to remove the endotoxins from the recombinant protein.

### Immunoblot analysis of rHCA59

Polyclonal antibodies against the rHCA59 protein were collected from SD rats by subcutaneous injection using 300 µg of rHCA59 protein mixed equally with Freund’s complete adjuvant. Rats were then injected three times with the same protein and Freund’s incomplete adjuvant at 1-week intervals 2 weeks after the first immunization. At 7 days after the last injection, the sera were collected and stored at -80 °C for use in later experiments. The sera from normal rats were also isolated as negative control. The anti-sera against *H. contortus* were harvested from experimentally infected goats and sera from normal goats were collected as a negative control. The recombinant HCA59 protein was separated on 12% SDS-PAGE and subsequently transferred to polyvinylidene difluoride (PVDF) membrane (Immobilon-PSQ, Millipore, Billerica, MA, USA) using a semi-dry system (Novablot, Hoefer, USA) in transfer buffer (39 mM glycine, 48 mM Tris, 20% methanol, 0.0375% SDS) at 1.5 mA/cm^2^ for 25 min. Next, the membrane was blocked with 5% (w/v) skim milk powder in TBST (TBS with 0.5% Tween-20) at 37 °C for 2 h, washed three times with TBST and incubated with anti-*H. contortus* sera from goats (1:300 dilutions) as the first antibody in the treatment group or normal goat sera in the negative control group (1:300 dilutions) for one night at 4 °C. The membranes were then washed and incubated with the secondary antibody, horseradish peroxidase (HRP)-conjugated rabbit anti-goat IgG (Santa Cruz Biotechnology, Dallas, TX, USA) in TBST (1:3000 dilutions), for 2 h at 37 °C. Next, the membranes were washed and immunoreactions were visualized within 3–5 min using a 3, 3-diaminobenzidine tetrahydrochloride kit (DAB; Sigma-Aldrich) according to the manufacturer’s instructions. Western blot analysis of HcESPs was conducted using the same procedures as for the rHCA59 protein above. During the process, the rat anti-sera against rHCA59 and normal rat sera were used as the first antibodies in the treatment group and the negative control group, respectively. The HRP-goat anti rat IgG (Beyotime, Jiangsu, China) was used as the secondary antibody.

### Preparation of goat PBMCs

Peripheral blood was collected from healthy donor male goats and PBMCs were isolated from the blood using the standard Ficoll-Hypaque (GE Healthcare, Munich, Germany) gradient centrifugation technique [20]. The fresh PBMCs were collected for the later cell proliferation, migration and nitric oxide production assays.

### Generation of goat monocyte-derived DCs

Monocyte-derived DCs (md-DCs) were isolated from PBMCs of native goats blood and identified as previously described [21, 22] with some modifications. Briefly, freshly collected PBMCs were incubated in 6-well cell culture plates (Corning Costar, Rochester, NY, USA) at 1.0 × 10^6^ cells/ml in 1640 RPMI medium (Gibco, Grand Island, NY, USA) with 10% fetal calf serum (Wisent, Jiangsu, China), 100 mg/ml streptomycin and 100 U/ml penicillin. After 12 h, the attached cells were separated by removing the supernatant and the non-attached cells [23], after which they were washed with phosphate-buffered saline (PBS). Next, the cells of the positive control group were stimulated using caprine granulocyte-macrophage colony-stimulating factor (20 ng/ml) (GM-CSF; Kingfisher, Washington, USA) and interleukin 4 (20 ng/ml) (IL-4; Kingfisher) for 3 days. Half of the cytokines were then added and cultured for another 3 days. Next, *E. coli* lipopolysaccharides (LPS; Sigma-Aldrich) were added at 1 μg/ml and incubated at 37 °C under 5% CO_2_ for 12 h. The cells of the experimental group were subsequently incubated with rHCA59 protein (20 μg/ml) for 3 days, after which half the quantity of the protein initially used was added and samples were cultured for 4 days as described earlier [24]. The cells in the control groups of pET-32a protein (20 μg/ml) and PBS (blank control) were also cultured using the same procedures as for the experimental group. During the experiment, cells were categorized by microscopy observation (Olymups, Shibuya, Japan) on days 1, 4 and 7 of the incubation period. On day 7, md-DCs from all groups were collected by gentle pipetting and centrifugation and used for further detection, while the md-DCs used for the binding experiment were generated from the positive group.

### Detection of rHCA59 binding to goat PBMCs and md-DCs

Immunofluorescence assays were conducted as previously described [25] to confirm the ability of rHCA59 to bind to goat PBMCs and md-DCs. Briefly, collected cells (1 × 10^6^) of PBMCs and md-DCs as described above were inoculated with rHCA59 for 1.5 h. Cells were then washed and permitted to settle onto poly-l-lysine treated glass slides for 30 min, after which they were fixed with 4% paraformaldehyde in PBS for 30 min at room temperature (RT). Next, cells were blocked with PBS containing 5% (w/v) BSA at 37 °C for 1 h. Subsequently, cells were cultured with primary antibodies (1:100 dilution), rat anti-rHCA59 sera or normal rat IgG (control) for 2 h and then incubated with secondary antibody goat anti-rat IgG (Beyotime) labeled with Cy3 (1:500 dilution) in the dark for 1 h. DAPI (Sigma, St. Louis, MO, USA) was subsequently added and samples were incubated for 5 min. Finally, the cells were washed, covered with a coverslip and dipped in Antifade Mounting Media (Beyotime). PBMCs and DCs were examined using two different confocal microscopes with laser scanners (PerkinElmer, Waltham, MA, USA; Nikon, Tokyo, Japan) and digital pictures were taken using the PerkinElmer or Nikon microscope software packages.

### Analysis of cell phenotypic markers of DCs by flow cytometry

Identification of DCs was conducted by flow cytometry (FACS) as previously described [24]. Briefly, DCs collected on day 7 were washed three times with PBS and then stained with antibodies. To detect the expression of CD14, cells were incubated with anti-bovine CD14 (Kingfisher, Washington, USA) and then APC-anti-mouse IgM (Biolegend, San Diego, CA, USA). To examine the quantity of CD172a, cells were cultured with anti-bovine CD172a (Kingfisher) and then FITC-anti-mouse IgG1 (Biolegend). To check the expression of CD11c, cells were incubated with anti-bovine CD11c (Kingfisher) and then RPE-anti-rat IgG1 (R&D Biotech, Minneapolis, CA, USA). To identify the quantity of CD80, cells were stained with RPE-mouse-anti-bovine co-stimulatory molecules CD80 (clone: IL159) (Bio-Rad, Hercules, CA, USA). Finally, cells stained with antibodies were analyzed using a FACS Caliber system (BD Bioscience, Franklin Lakes, NJ, USA).

### Detection of key genes in WNT pathway related to md-DCs’ differentiation and maturation

Total DNA-free RNA of md-DCs was isolated from cells (2.5 × 10^6^) of rHCA59-treated and control groups and harvested on day 7 using Trizol reagent (Invitrogen) and the previously described method [13]. The quantity and reliability of extracted RNA were checked by spectrophotometry using a biophotometer (Eppendorf, Berlin, Germany). The absorption ratios (OD260/OD280) of all samples were between 1.8 and 2.0. The cDNA was then prepared in a 20 μl reaction mixture using 1 μg of total RNA per reaction. Thermo-Script RT and Oligo (dT) (Invitrogen, Foster, CA, USA) were used to synthesize cDNA according to the manufacturer’s specifications. The cDNA of rHCA59-treated and control groups were used for examination of the key genes of β-catenin, CK2, DVL and APC for the WNT pathway by quantitative real-time reverse transcriptase polymerase chain reaction (qPCR). The reaction for the detection of key genes was conducted in a total volume of 20 μl containing 2 μl of 2× SYBR qPCR Master Mix, 0.4 μl of each primer (10 μM), 0.4 μl of 50× ROX Reference Dye1, 2 μl of cDNA and 6.8 μl of ddH_2_O. The primers used for amplification of the key genes investigated are listed in Additional file 1: Table S1. Data are representative of three independent experiments.

### Detection of cytokines level

The IL-2, IL-4, IL-10, IL-17, IFN-γ and TGF-β1 secretion levels of PBMCs were detected by qPCR. Briefly, PBMCs freshly isolated as above were stimulated with ConA (10 μg/ml) and serial concentrations of rHCA59 (10, 20 and 40 µg/ml), pET-32a protein and the same volume of PBS for 48 h at 37 °C under 5% CO_2_. Subsequently, cells were collected and the total RNA was extracted using an RNeasy Mini Kit (Qiagen, Hilden, Germany) as previously described [13]. The primers used for qPCR are shown in Additional file 2: Table S2. The data are representative of three independent experiments.

### Cell proliferation assay

The cell proliferation assay was conducted as previously described [26]. Briefly, suspensions of fresh PBMCs isolated as above (1 × 10^6^ cells/ml) were activated with ConA (10 µg/ml), serial concentrations of rHCA59 (10, 20 and 40 µg/ml), pET-32a protein, and the same volume of control buffer (PBS), then cultured under 5% CO_2_ at 37 °C for 72 h. Next, 10 μl of CCK-8 reagents (Beyotime) were added to each well of the plates for 4 h before measuring the absorbance values at 450 nm (OD450) in a microplate reader (Thermo Scientific, Minneapolis, MN, USA). Data are representative of one of three independent experiments.

### Cell migration assay

The cell migration experiment was conducted utilizing a Transwell system (Merck-Millipore, Boston, MA, USA), which allowed PBMCs to migrate through the polycarbonate membrane with an 8 µm pore size [27]. Cells were cultured with different concentrations of rHCA59 (10, 20 and 40 µg/ml), pET-32a protein, and an equal volume of PBS (control buffer). Each experiment was performed in triplicate.

### Nitric oxide production assay

Fresh PBMCs (1 × 10^6^ cells/ml) isolated as above were washed two times with PBS and incubated in 96-well plates in DMEM. The production of nitric oxide (NO) was then examined as intracellular nitrite in PBMCs by a Griess assay [28] using a Total Nitric Oxide Assay Kit (Beyotime). Absorbance values at 540 nm (OD540) of the colored solution were measured using a plate reader (Bio-Rad), then transformed to micromoles per liter (μmol/l) using a standard curve that had been produced by the addition of 0–80 μmol/l sodium nitrite to fresh culture media. The individual experiment was performed three times.

### Cell phagocytosis assay

Fresh monocytes (1 × 10^6^ cells/ml) were separated from PBMCs by their adherence to the cell plate as described above, then cultured with serial concentrations of rHCA59 (10, 20 and 40 µg/ml), pET-32a protein, and same volume of control buffer (PBS) and cultured with 5% CO_2_ for 48 h at 37 °C. Next, cells were collected, washed and transferred to a 1.5-ml tube in 100 µl PBS and then mixed with 100 µl FITC-dextran in the dark for 1 h at 37 °C before testing using flow cytometry (BD Bioscience). The ratio of the mean fluorescence intensity (MFI) of each group to the PBS group was utilized as the phagocytic index. Three independent experiments were performed.

### Statistical data analysis

Statistical analyses were conducted using the GraphPad 6.0 software (GraphPad Prism, San Diego, CA, USA). All of the data obtained from the above experiments were reported as the means ± SD. Differences between groups were identified by ANOVA followed by Tukey’s *post-hoc* test, with *P* < 0.05, *P* < 0.01 and *P* < 0.001. The FACS data were analyzed using FlowJo (version 10, Franklin Lakes, NJ, USA) software.

## Results

### Molecular cloning of HCA59 gene

The fragments of HCA59 genes (426 bp) cloned into vectors pMD-19T and pET-32a were confirmed by restriction enzyme digestion with *BamH*I and *Hind*III and sequence analysis. The results indicated that the gene was successfully cloned into the vectors (Additional file 3: Figure S1).

### Expression, purification and western blot analysis of rHCA59

**Fig. 1:**
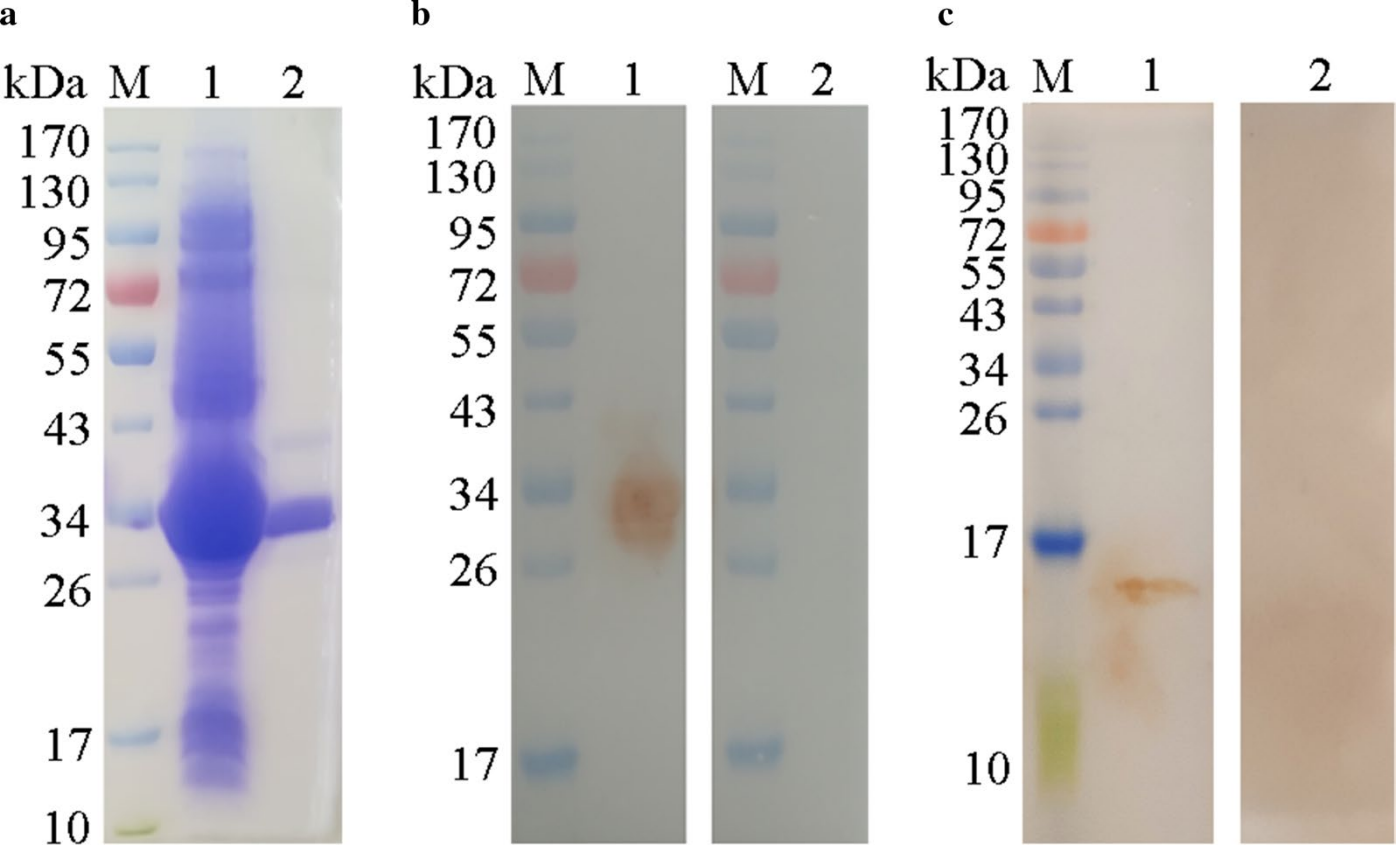
Expression, purification and Western blot analysis of rHCA59 protein. Lane M: standard protein molecular weight marker. **a** rHCA59 protein was induced with 1 mM IPTG. Lane 1: expression of recombinant protein rHCA59 vector before purification; Lane 2: purified rHCA59 protein. **b** Western blot of rHCA59 protein. Lane 1: recombinant protein HCA59 was recognized by goat anti-*H. contortus* sera; Lane 2: membrane incubated with normal goat sera (as control). **c** Western blot of total HcESPs. Lane 1: HcESPs were detected by rat anti-rHCA59 protein antibodies; Lane 2: membrane incubated with normal rat sera (as control)

The recombinant protein of HCA59 was expressed and purified as His tagged fusion protein. The purified recombinant protein was resolved on 12% SDS-PAGE and had a molecular weight of about 33 kDa (Fig. 1a). The calculated molecular mass of the HCA59 protein was its original size of 16 kDa after reduction of the His tagged fusion protein of the pET-32a vector (17 kDa). Western blot analysis showed that the recombinant HCA59 could be recognized by sera from goats infected with *H.contortus*, while native HCA59 protein from HcESPs could be recognized by antibodies generated against rHCA59 protein (Fig. 1b, c, Lane 1). The molecular mass of the native HCA59 protein was 16 kDa, which was the same molecular weight as that of the rHCA59 after reduction from pET-32a protein. However, no protein was detected with normal goat sera or normal rat sera (Fig. 1b, c, Lane 2). These findings suggest that protein rHCA59 had good antigenic characteristics and could be recognized by the host immune system.

### Validation of rHCA59 binding with PBMCs and md-DCs

The bindings of rHCA59 to goat PBMCs and md-DCs are shown in Additional file 4: Figure S2a (PBMCs) and Additional file 4: Figure S2b (DCs). The cells subjected to secondary antibody labelled with Cy3 are presented in red, the nuclei of the cells are shown in blue, and combined pictures of protein binding show merged red and blue. However, the cells of the control group did not show any fluorescence (Additional file 4: Figure S2a2, a4, a6; Additional file 4: Figure S2b2, b4, b6). The dense concentration of red around the PBMCs and md-DCs indicated that rHCA59 could bind strongly to the PBMCs and md-DCs surfaces.

### Effect of rHCA59 on maturation of md-DCs

Mature md-DCs were semi-suspended or suspended in the medium with a round appearance and had one or more dendritic processes. On day 7 of the experiment, microscope observation revealed that cells incubated with GM-CSF+IL-4+LPS (positive group) were large and irregularly shaped, and that they occurred as single cells or clusters of cells with pseudopodia of noticeable length, all of which were characteristics expected for md-DCs (Additional file 5: Figure S3a). Cells incubated with rHCA59 presented the same features as the positive group, while only a few of the cells cultured with pET-32a protein or PBS presented the characteristics of the positive group (Additional file 5: Figure S3b–d).

**Fig. 2:**
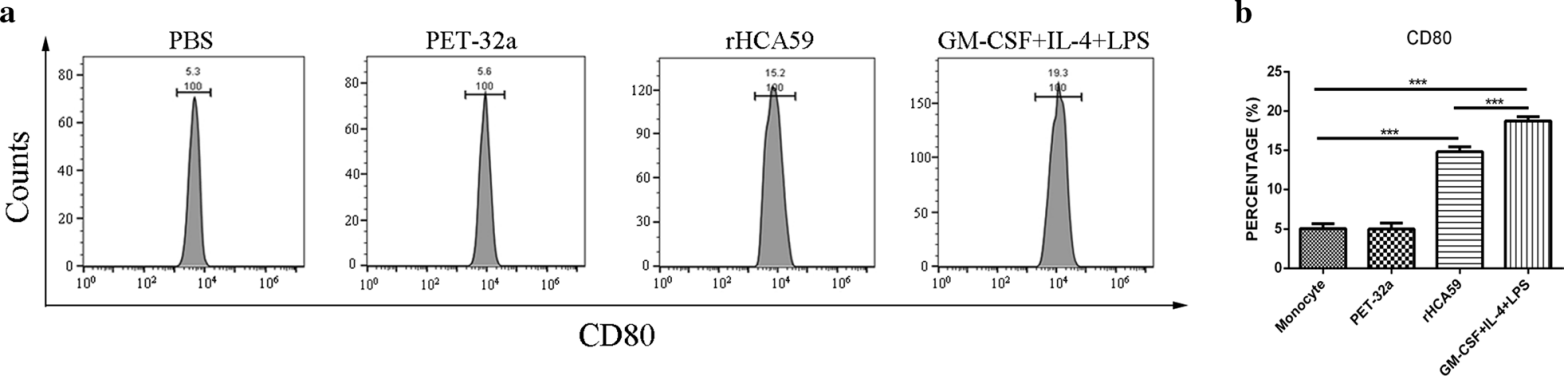
rHCA59 increases the expression of surface marker of CD80 in md-DCs. **a** Percentage of CD80 in md-DCs as determined by flow cytometry. **b** Different stimulations affect the proportion of CD80. Results shown here are from an independent experiment that is representative of three independent experiments (*P < 0.05, **P < 0.01, ***P < 0.001)

**Fig. 3:**
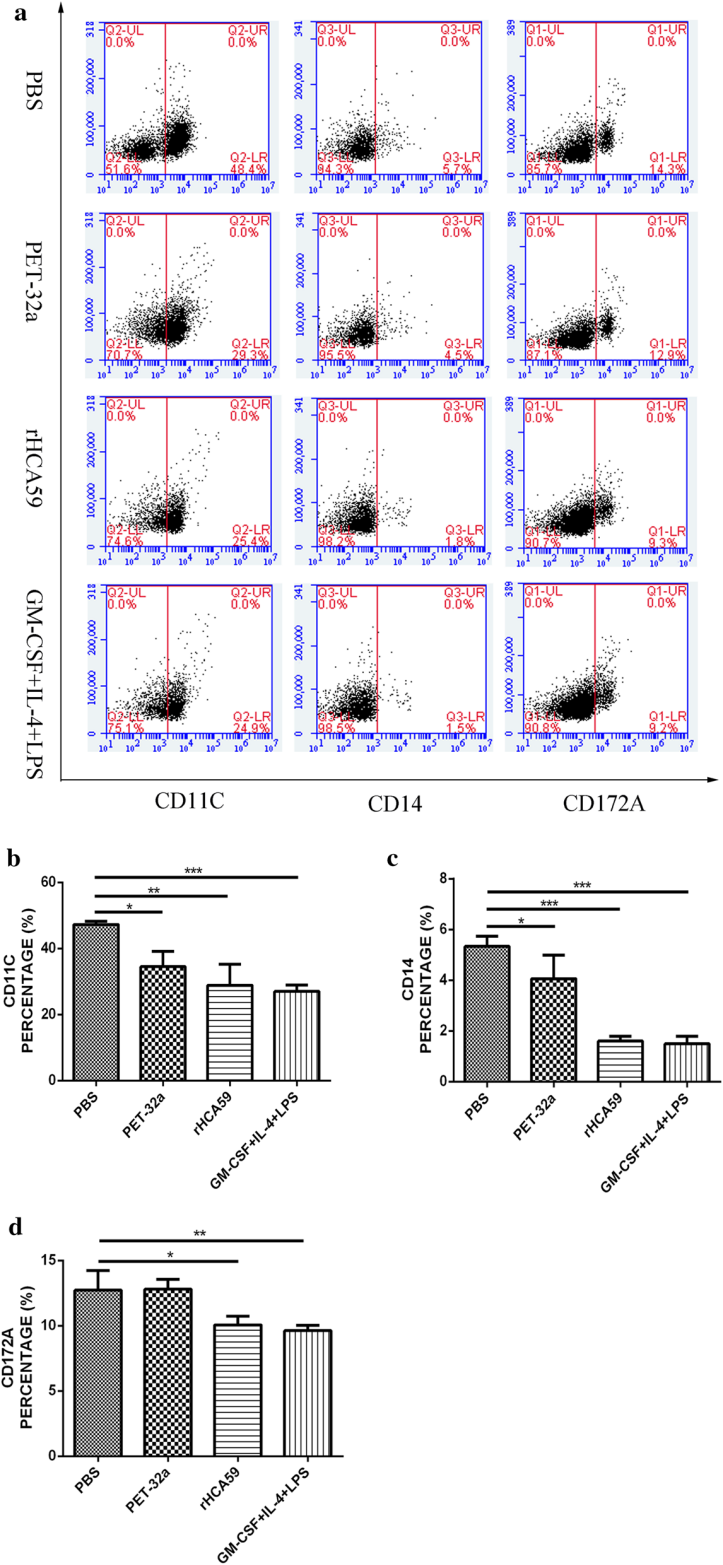
Effects of rHCA59 on the expression of surface markers of CD11c, CD14 and CD172a in md-DCs. Samples were analyzed by flow cytometry. **a** Dot plot analysis of monocytes treated with PBS, pET-32a protein, rHCA59 and GM-CSF+IL-4+LPS at day 7. **b** Percentage of CD11c in different groups. **c** Percentage of CD14 in different groups. **d** Percentage of CD172a in different groups. Data are presented as the mean ± SEM and representative of triplicate experiments (*P < 0.05, **P < 0.01, ***P < 0.001)

Cell phenotypes were also evaluated by flow cytometry on day 7. The results showed that the percentages of CD80 were significantly increased in the rHCA59 protein group (ANOVA: *F*_(3,8)_ = 344.0, *P* < 0.0001) and GM-CSF+IL-4+LPS group (ANOVA: *F*_(3,8)_ = 344.0, *P* < 0.0001) when compared with PBS and pET-32a protein groups (Fig. 2). These findings showed that rHCA59 could greatly increase the expression of CD80 and enhance the percentages of DC cells. To further confirm these findings, the expressions of CD14, CD11c and CD172a were also tested. The positive cells displayed a relatively lower expression level of CD14, CD11c and CD172a relative to the PBS group (ANOVA: CD14: *F*_(3,8)_ = 37.11, *P* < 0.0001; CD11c: *F*_(3,8)_ = 15.15, *P* < 0.0001; CD172a: *F*_(3,8)_ = 10.00, *P* < 0.01). The rHCA59 protein group presented a similar trend as the positive group (ANOVA: CD14: *F*_(3,8)_ = 37.11, *P* < 0.0001; CD11c: *F*_(3,8)_ = 15.15, *P* < 0.01; CD172a: *F*_(3,8)_ = 10.00, *P* < 0.05) (Fig. 3). However, the pET-32a protein group did not decrease the expression of CD172a when compared with the PBS group (ANOVA: CD172a: *F*_(3,8)_ = 10.00, *P* = 0.9486) (Fig. 3d). These findings also indicated that rHCA59 could increase the percentages of DC cells.

### rHCA59 activated the WNT pathway related to md-DCs differentiation and maturation

**Fig. 4:**
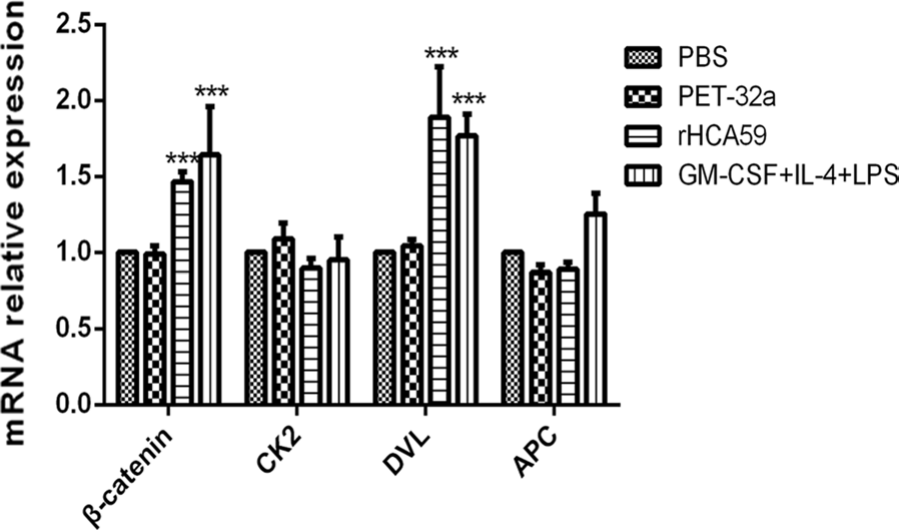
Effects of rHCA59 on the expression profiles of candidate genes belonging to the WNT signal pathway in goat DCs. Total RNA was extracted from goat md-DCs and the expression of key genes (β-catenin, DVL, CK2 and APC) was assayed by qPCR. Symbolic data from one independent experiment with methodical triplicates representative of three independent experiments are presented (*P < 0.05, **P < 0.01, ***P < 0.001)

The key genes of β-catenin, CK2, DVL and APC of the WNT pathway, were evaluated by qPCR. The results showed that β-catenin and DVL presented significantly elevated expression when cells were incubated with GM-CSF+IL-4+LPS (ANOVA: β-catenin: *F*_(9,32)_ = 10.42, *P* < 0.0001; DVL: *F*_(9,32)_ = 10.42, *P* < 0.0001) and rHCA59 (ANOVA: β-catenin: *F*_(9, 32)_ = 10.42, *P* < 0.001; DVL: *F*_(9, 32)_ = 10.42, *P* < 0.0001). However, the expression of CK2 and APC in the GM-CSF+IL-4+LPS group (ANOVA: CK2: *F*_(9,32)_ = 10.42, *P* = 0.6847; APC: *F*_(9,32)_ = 10.42, *P* = 0.0557) and the rHCA59 protein group (ANOVA: CK2: *F*_(9,32)_ = 10.42, *P* = 0.3246; APC: *F*_(9,32)_ = 10.42, *P* = 0.2903) did not show obvious changes relative to the pET-32a protein and PBS groups (controls) (Fig. 4).

### Protein rHCA59 modulated cytokine secretion by PBMCs

**Fig. 5:**
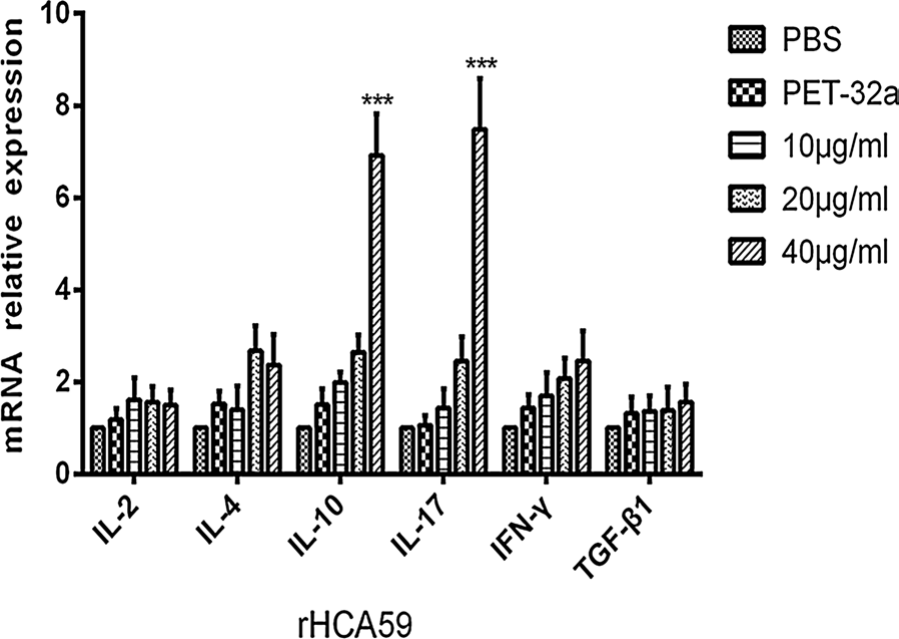
Analysis of the levels of production of multiple cytokines of PBMCs by qPCR *in vitro*. PBMCs were cultured with pET-32a protein, PBS (control) and serial concentrations of rHCA59 for 72 h. Data are representative of three independent experiments and the values presented here are the means ± SEM (*P < 0.05, **P < 0.01, ***P < 0.001)

A qPCR assay was conducted to assess the cytokine production by PBMCs that had been treated with rHCA59. As shown in Fig. 5, the expression of IL-10 and IL-17 increased significantly when PBMCs were cultured with 40 µg/ml rHCA59, while the expression of IL-2, IL-4, IFN-γ and TGF-β1 did not show obvious changes when compared with the pET-32a protein and PBS groups [ANOVA: IL-2 (10 μg/ml: *F*_(20,60)_ = 7.249, *P* = 0.4241; 20 μg/ml: *F*_(20,60)_ = 7.249, *P* = 0.5059; 40 μg/ml: *F*_(20,60)_ = 7.249, *P* = 0.6939), IL-4 (10 μg/ml: *F*_(20,60)_ = 7.249, *P* = 0.8084; 20 μg/ml: *F*_(20,60)_ = 7.249, *P* = 0.0554; 40 μg/ml: *F*_(20,60)_ = 7.249, *P* = 0.2875), IL-10 (10 μg/ml: *F*_(20,60)_ = 7.249, *P* = 0.3555; 20 μg/ml: *F*_(20,60)_ = 7.249, *P* = 0.0589; 40 μg/ml: *F*_(20,60)_ = 7.249, *P* < 0.0001), IL-17 (10 μg/ml: *F*_(20,60)_ = 7.249, *P* = 0.4729; 20 μg/ml: *F*_(20,60)_ = 7.249, *P* = 0.0574; 40 μg/ml: *F*_(20,60)_ = 7.249, *P* < 0.0001), IFN-γ (10 μg/ml: *F*_(20,60)_ = 7.249, *P* = 0.6148; 20 μg/ml: *F*_(20,60)_ = 7.249, *P* = 0.2518; 40 μg/ml: *F*_(20,60)_ = 7.249, *P* = 0.2010) and TGF-β1 (10 μg/ml: *F*_(20,60)_ = 7.249, *P* = 0.9338; 20 μg/ml: *F*_(20,60)_ = 7.249, *P* = 0.9089; 40 μg/ml: *F*_(20,60)_ = 7.249, *P* = 0.7540)].

### rHCA59 increased cell proliferation of goat PMBCs

**Fig. 6:**
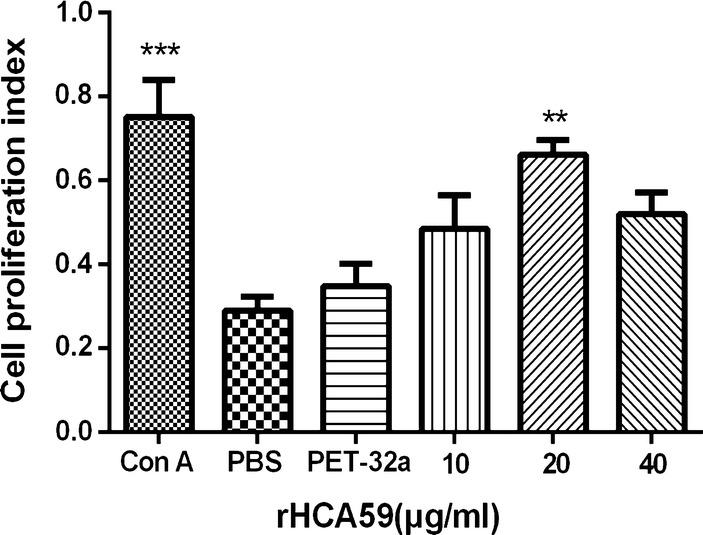
Effects of different concentrations of rHCA59 on PBMCs proliferation. Cells were treated with ConA (10 µg/ml), PBS (control buffer), pET-32a protein and serial concentrations of rHCA59 with 5% CO_2_ at 37 °C. The proliferation assay was implemented by CCK-8 incorporation after 72 h and the cell proliferation index was analyzed based on comparison to the OD450 values in control group as 100% (**P < 0.01, ***P < 0.001)

A cell proliferation assay was conducted to assess the effects of rHCA59 on cell proliferation using a CCK-8 reagent kit. The results illustrated that cell proliferation was obviously increased in cells cultured with 20 μg/ml of rHCA59 when compared with the pET-32a protein and PBS groups (ANOVA: 10 μg/ml: *F*_(5,13)_ = 7.645, *P* = 0.2288; 20 μg/ml: *F*_(5,13)_ = 7.645, *P* < 0.01; 40 μg/ml: *F*_(5,13)_ = 7.645, *P* = 0.0810; ConA: *F*_(5,13)_ = 7.645, *P* < 0.001) (Fig. 6).

### rHCA59 decreased cell migration of goat PMBCs

**Fig. 7:**
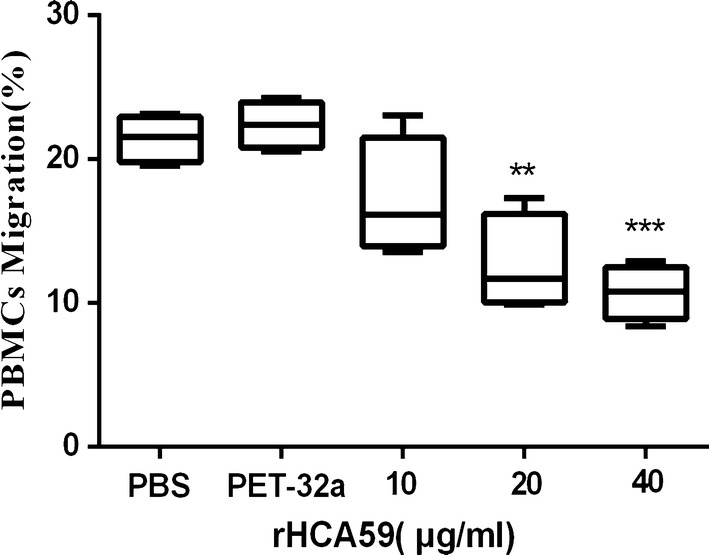
Different concentrations of rHCA59 protein affect PBMCs migration. PBMCs were treated with PBS (control buffer), pET-32a protein, and different concentrations of rHCA59. Results are presented as a whisker plot (max and min values), with the boxes covering 50% of the values. The median is indicated by the horizontal bar. Results confirmed here are based on one independent experiment (*n* = 4) and are demonstrative of three independent experiments (**P < 0.01, ***P < 0.001)

In the present study, a cell migration assay was conducted using a Transwell system to evaluate the effects of rHCA59 on cell migration. The results demonstrated that cell migration decreased obviously in response to treatment with 20 and 40 μg/ml of rHCA59 when compared with the pET-32a protein and PBS groups (ANOVA: 10 μg/ml: *F*_(4,15)_ = 14.34, *P* = 0.1038; 20 μg/ml: *F*_(4,15)_ = 14.34, *P* < 0.01; 40 μg/ml: *F*_(4,15)_ = 14.34, *P* < 0.001) (Fig. 7).

### rHCA59 increased the production of nitric oxide generated by goat PMBCs

**Fig. 8:**
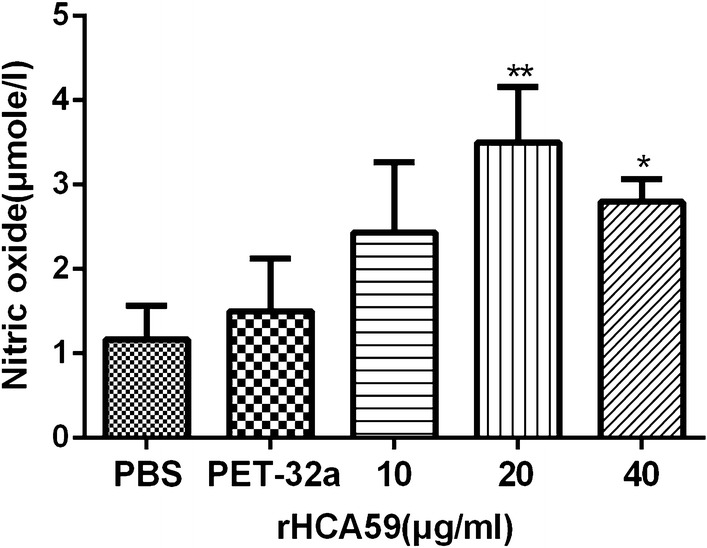
Effects of rHCA59 on PBMCs intracellular nitric oxide production. PBMCs were cultured with PBS (control buffer), pET-32a protein, and different concentrations of rHCA59 at 37 °C under 5% CO_2_. The NO concentrations in the PBMCs were determined by Griess assay. The data are presented as the means ± SEM and are representative of triplicate experiments (*P < 0.05, **P < 0.01)

A nitric oxide (NO) assay was conducted to measure the NO production by PBMCs cultured with serial concentrations of rHCA59 using a total nitric oxide assay kit. The results showed that treatment with rHCA59 at 20 and 40 μg/ml significantly prompted the NO production by PBMCs when compared with the pET-32a protein and PBS groups (ANOVA: 10 μg/ml: *F*_(4,10)_ = 7.794, *P* = 0.1953; 20 μg/ml: *F*_(4,10)_ = 7.794, *P* < 0.01; 40 μg/ml: *F*_(4,10)_ = 7.794, *P* < 0.05) (Fig. 8).

### Monocyte phagocytosis assay

**Fig. 9:**
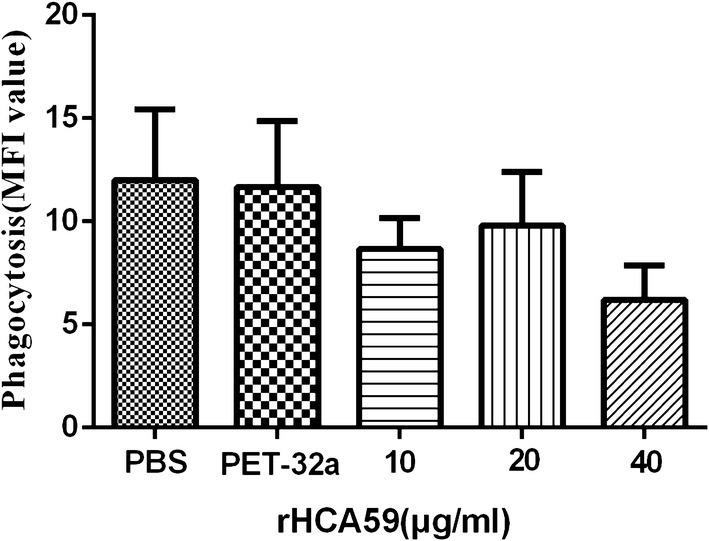
rHCA59 did not obviously affect the phagocytic capability of goat monocytes. Monocytes were treated with serial concentrations of rHCA59, pET-32a protein, and PBS (control buffer) for 48 h, then cultured with FITC-dextran at 37 °C for 1 h before they were collected. Flow cytometry was used to assess the FITC-dextran internalization of monocytes and the phagocytic index is shown as a reference. Data are representative of independent experiments conducted in triplicate (*P < 0.05, **P < 0.01, ***P < 0.001)

In this study, a monocyte phagocytosis assay was conducted to test the phagocytosis abilities of monocytes treated with serial concentrations of rHCA59 and the phagocytic ability of monocytes was checked by evaluation of FITC-dextran uptake. The results confirmed that the ability of monocytes treated with rHCA59 to swallow FITC-dextran decreased, but not significantly (ANOVA: 10 μg/ml: *F*_(4,10)_ = 2.500, *P* = 0.2142; 20 μg/ml: *F*_(4,10)_ = 2.500, *P* = 0.4763; 40 μg/ml: *F*_(4,10)_ = 2.500, *P* = 0.0580) (Fig. 9).

## Discussion

Many molecules of ESPs of *H. contortus* have been recognized and shown to play vital roles in host-parasite interactions by adjusting host immune responses against parasitic infections [29]. HCA59 belongs to the TLS1 family found in nucleoplasm and cytosol and has already been examined in human and drosophila germlines [11, 30]. During *H. contortus* infection, HCA59 of the parasite was first found to bind to the PBMCs of goats at larval stages 4 and 5 (L4 and L5) of the nematode *in vitro* [10]. These findings suggested that HCA59 of *H. contortus* participated in interactions between the nematode and the host and might regulate the host immune responses to parasite infection. Thus, in this study, the immunomodulatory effects of HCA59 of *H. contortus* on dendritic cells and PBMCs of goats were investigated *in vitro*.

DCs are professional antigen sensing and presenting cells that serve as the key linkage between innate and adaptive immunities. The pathogen components recognized by immature DCs promote DCs maturation and activation and initiate T cell immune responses during infection. Mature DCs present antigen to antigen specific T cells and modulate T cells differentiation toward Th1, Th2, Th17 or regulatory T (Treg) cells [15, 31]. The high expression of CD80 is usually considered a key sign of DC maturity [32]. In the present study, the results indicated that rHCA59 protein enhanced the expression of CD80 of md-DCs dramatically (Fig. 2b), suggesting that rHCA59 of *H. contortus* could promote the mature DCs of the host. The expressions of CD14, CD11c and CD172a in md-DCs has also been reported to be lower than in monocytes [22, 33, 34]. In this study, cells cultured with rHCA59 expressed relatively lower levels of CD14, CD11c and CD172a than monocytes, similar to the results observed in cells treated with GM-CSF+IL-4+LPS (positive group) (Fig. 3). However, cells cultured with pET-32a protein did not show similar changes. These findings indicated that rHCA59 could induce monocytes to develop into mature DCs.

It has been reported that the WNT/β-catenin pathway might regulate functions of DCs and that the WNT/β-catenin pathway was activated the maturation of DCs [17, 21]. A previous study demonstrated that during the WNT/β-catenin pathway activation processes, the DVL (the receptor of membrane protein Frizzled) is activated first. The increased DVL protein then results in the destruction of the complex consisting of scaffolding proteins, including APC, CK proteins and GSK3β, leading to GSK3β phosphorylation. The phosphorylated GSK3β activates the β-catenin, then the increases entry of the β-catenin protein into the nucleus to participate in the process of transcription. During the process, the quantities of APC and CK molecules do not change after separation from the scaffolding protein complex because they do not partake in the following reactions [35–37]. In this investigation, the expression of DVL, CK2, APC and β-catenin in the WNT pathway was examined. The results showed that, when compared to the PBS and pET-32a protein groups, the mRNA expression of DVL, β-catenin in rHCA59 and the GM-CSF+IL-4+LPS groups were upregulated, but the CK2 and APC did not change (Fig. 4). The change profiles of the key genes were in accordance with those that in the activation processes of the WNT/β-catenin pathway. These findings indicted that rHCA59 could activate the WNT pathway and promote the differentiation and maturation of md-DCs. However, during the maturation of DCs, some other important pathways, such as MAPK and NFĸB [38, 39], as well as some complex mechanisms, such as the interactions between the MAPK and WNT pathways, were also involved [21]. The effects of rHCA59 on these complex mechanisms should be explored in future studies.

Cytokines play significant roles in immune responses [40, 41]. The Th2 immunity related to the secretion of IL-4 is usually considered to be the major immune mechanism against helminthes, including *H. contortus* [42]. In this study, the effects of rHCA59 on the levels of the typical cytokines Th1 (IL-2, IFN-γ), Th2 (IL-4), pro-inflammatory IL-17 and Treg cytokines (TGF-β1, IL-10) were checked. The results showed that rHCA59 significantly increased the secretions of IL-17 and IL-10 of goat PBMCs, but had no effect on the other cytokine secretions (Fig. 5). The IL-17 cytokine is produced by Th17 effectors cells and has been shown to be associated with pathogenesis and inflammatory responses of many parasites [43, 44]. The enhanced level of IL-17 indicated that rHCA59 might play roles in the pathogenesis of *H. contortus*. IL-10 and TGF-β1 are produced by Treg cells and are usually considered to function in immune suppression [45, 46]. The increase of IL-10 in the present study suggested that HCA59 of *H. contortus* might function in the immune evasion of the nematode. In previous studies, rHcES-24, rHcFTT-2 and rHcARF1, ESP components of *H. contortus*, and the total ESP of this nematode were shown to increase the production of IL-17 or IL-10 [26, 47–49]. These results, together with those of the present study, suggest that more molecules of the ESP of *H. contortus* are involved in the pathogenesis and immune evasions of this parasite. However, in the present study, rHCA59 only enhanced the production of IL-10, but not both TGF-β1 and IL-10. Future studies should be conducted to further investigate the mechanisms involved.

Hook worm crude antigen has been reported to harm cell proliferation and reduce the number of Treg cells [50] and that the immune responses caused by helminths infection could be changed by adjusting the proliferation of immune cells [51]. In previous studies, suppressive regulation of rHcES-24 and rHcFTT-2 was found during cell proliferation experiments *in vitro* [47, 48]. Contrary to the former results, promotional activity of rHCA59 at a concentration of 20 μg/ml was observed on PBMC proliferation (Fig. 6). These mechanisms involved in these promoting effects should be further researched.

Helminths could actively stimulate immunocyte (lymphocytes; eosinophil) migrations to the sites of infection and caused tissue damage to worms found inside the host [52]. Previous studies suggested that HcESPs and rHcES-24 could significantly increase the migration of PBMCs [26, 48]. Conversely, in this study, cell migration declined as the level of HCA59 protein increased (Fig. 7). The actual mechanisms involved in this suppressive adjustment of cell trafficking requires further study.

Nitric oxide has been examined for its roles in immunity and inflammation in many cell lines [53]. The enhancement of NO production could help host cells kill pathogens and might also increase the inflammatory responses [54, 55]. In this study, a significant increase in NO production at 20 and 40 μg/ml of rHCA59 (Fig. 8) was observed, suggesting that rHCA59 participated in the immune regulation of NO in goat PBMCs.

Monocytes in the blood are a source of macrophages in tissues, and it has been reported that macrophages could directly kill certain intracellular parasites, such as *Leishmania* and *Toxoplasma* [56, 57], *via* phagocytosis after activation by some cytokines such as IFN-γ [58]. In this study, rHCA59 exerted certain inhibitory effects on the phagocytosis of monocytes, but these were not obvious (Fig. 9). These findings indicated that rHCA59 did not affect the phagocytosis of PBMCs of goats, in accordance with the finding that it did not increase the secretion of IFN-γ.

Proteins in eukaryotic cells are commonly modified for their mature and biological functions. The most common protein modifications in eukaryotes include glycosylation and phosphorylation [59–61]. In this study, the phosphorylation and glycosylation of HCA59 of *Haemonchus contortus* were analyzed using the NetPhos 3.1 Server (http://www.cbs.dtu.dk/services/NetPhos/), big-PI Predictor (http://mendel.imp.ac.at/sat/gpi/gpi_server.html) and YinOYang 1.2 Server (http://www.cbs.dtu.dk/services/YinOYang/). The results showed that the protein had 12 phosphorylation sites, four O-GlcNAc sites and no GPI modification sites. In general, the molecular mass of protein might increase after modification. In the present study, the molecular weight of native HCA59 from ESP was 16 kDa, which was identical to that of rHCA59 after reduction from pET-32a protein (Fig. 1c). Further studies are needed to determine the mechanisms responsible for changes in protein molecular mass.

Generally, the functions of the recombinant proteins expressed by the prokaryotic system might be modified when compared to native proteins because of its lack of glycosylation and phosphorylation. However, it has been reported that *Escherichia coli*-expressed proteins shared the same biological functions as the native proteins [62, 63]. In this study, the recombinant protein of HCA59 was obtained by the expression in *Escherichia coli* and rHCA59 protein showed good biological functions with respect to modulating the functions of PBMCs and the differentiation and maturation of monocyte-derived dendritic cells of goats *in vitro*. Future studies should investigate whether functional differences exist between rHCA59 and native HCA59 protein.

## Conclusions

This study showed that HCA59 is an important and active protein of HcESPs that significantly promoted the differentiation and maturation of DCs, increased IL-10 and IL-17 levels and cell proliferation, and decreased PBMCs migration. The results suggest that the promotion of differentiation and maturation of DCs results in T cells differentiation toward Th17 and Treg, but not Th1 and Th2. The increased IL-10 and IL-17 levels and decreased PBMCs migration suggest that HCA59 mainly functions in the pathogenesis and immune evasions of this parasite. These results not only improve our understanding of the functions of HCA59 and ESP of this parasite, but also help clarify the mechanisms involved in immune evasion by *H. contortus* during host-parasite interactions. Our results also suggest that HCA59 might be a candidate molecule for development of a vaccine against *H. contortus* infection.

## Additional files

**Additional file 1: Table S1.** Primer sequences of target genes related to WNT pathway.

**Additional file 2: Table S2.** Primer sequences of cytokines secreted by PBMCS.

**Additional file 3: Figure S1.** Double digestion analysis of HCA59. Lane M: DNA marker. **a** HCA59-19T plasmid digested by *BamH*I and *Hind*III. Lane 1: double digestion products with pMD19-T vector (2692 bp) and HCA59 (426 bp). **b** HCA59-pET32a plasmid digested by *BamH*I and *Hind*III. Lane 1: double digestion products with pET-32a vector (5900 bp) and HCA59 (426 bp).

**Additional file 4: Figure S2.** An immunofluorescence assay was conducted to determine if rHCA59 could bind to goat PBMCs (**a**) and md-DCs (**b**). Localization was performed by incubating DCs or PBMCs with rat anti-rHCA59 IgG or negative rat IgG (control). **a1**, **a2**, **b1**, **b2** Staining of the target proteins (red) was visualized by Cy3-conjugated secondary antibody. **a3**, **a4**, **b3**, **b4** Nuclei of the corresponding cells were visualized by DAPI (blue) staining. **a5**, **a6**, **b5**, **b6** Merged, overlap of the red and blue channels. No red fluorescence was observed in the control group. *Scale-bars*: **a**, 10 μm; **b**, 5 μm.

**Additional file 5: Figure S3.** Generation of monocyte-derived dendritic cells from goat PBMCs. Morphology of cells was viewed by optical microscopy at 400× magnification. **a** Cells supplemented with GM-CSF+IL-4+LPS (positive group). **b** Cells treated with rHCA59 (tested group). **c** Cells treated with pET-32a protein (negative control). **d** Cells treated with PBS (blank control).

## Abbreviations

HCA59: hepatocellular carcinoma-associated antigen 59
rHCA59: recombinant protein of HCA59
HcESPs: excretory/secretory products of *Haemonchus contortus*
DCs: dendritic cells
IFA: immunofluorescence assay
NO: nitric oxide
PBMC: peripheral blood mononuclear cells
HCA587: hepatocellular carcinoma-associated antigen 587
MAPKs: mitogen-activated protein kinases
CDS: conserved domain sequences
ORF: open reading frame
LB: Luria Bertini
IPTG: Isopropyl-β-d-thiogalactopyranoside
SDS-PAGE: sodium dodecyl sulfate-polyacrylamide gel electrophoresis
Ni-NTA: Ni2+-nitrilotriacetic acid
PVDF: polyvinylidene difluoride
DAB: 3, 3-diaminobenzidine tetrahydrochloride
md-DCs: monocyte-derived DCs
GM-CSF: granulocyte-macrophage colony-stimulating factor
IL-4: interleukin 4
LPS: lipopolysaccharides
qPCR: quantitative real-time reverse transcriptase polymerase chain reaction
IL-2: interleukin 2
IL-10: interleukin 10
IL-17: interleukin 17
TGF-β1: transforming growth factor-β1
IFN-γ: interferon-γ
ConA: concanavalin A
PBS: phosphate-buffered saline
RT: room temperature
DAPI: 2-(4-amidinophenyl)-6-indole carbamidinedihydrochloride
BSA: Bovine Serum Albumin
CCK-8: Cell Counting Kit-8
MFI: mean fluorescence intensity
