# Supplementary material for: Hepatocellular carcinoma-associated antigen 59 of Haemonchus contortus modulates the functions of PBMCs and the differentiation and maturation of monocyte-derived dendritic cells of goats in vitro
Source: Parasit Vectors. 2019 Mar 14;12:105. doi: 10.1186/s13071-019-3375-1 (PMC6416944; doi:10.1186/s13071-019-3375-1)
Supplement: Supplementary file 1 — Additional file 1: Table S1. Primer sequences of target genes related to WNT pathway. [file 13071_2019_3375_MOESM1_ESM.docx]

**Table S1. Primer sequences for real-time PCR**

| Gene Name | Forward 5 → 3 | Reverse 5 → 3 | Amplification size (b p) |  | Amplification efficiency (%) * |
| --- | --- | --- | --- | --- | --- |
| beta-actin | CACCACACCTTCTACAAC | TCTGGGTCATCTTCTCAC | 106 |  | 96.964 |
| β-catenin | AACTTGCCACACGTGCAATC | CACCATCTGAGGAGAACGCA | 146 |  | 100.923 |
| CK2 | GAAGTCTAATCAGCCGGCCA | TCAACGTCCAAGTCACACGA | 94 |  | 90.492 |
| APC | CCATGCATTGGCAGTTAACCA | CCGTATGCAAGGCAGTTCAC | 137 |  | 93.912 |
| DVL | CACCCACGCACACATACAC | GGATGACGCTCCCAACTAGATA | 93 |  | 106.288 |

^*^ Amplification efficiency (%) = (10^-1/slope^ -1) ×100
