# Supplementary material for: Hepatocellular carcinoma-associated antigen 59 of Haemonchus contortus modulates the functions of PBMCs and the differentiation and maturation of monocyte-derived dendritic cells of goats in vitro
Source: Parasit Vectors. 2019 Mar 14;12:105. doi: 10.1186/s13071-019-3375-1 (PMC6416944; doi:10.1186/s13071-019-3375-1)
Supplement: Supplementary file 2 — Additional file 2: Table S2. Primer sequences of cytokines secreted by PBMCS. [file 13071_2019_3375_MOESM2_ESM.docx]

| Gene Name | Forward 5 → 3 | Reverse 5 → 3 | Amplification size (b p) | Amplification efficiency (%) * |
| --- | --- | --- | --- | --- |
| beta-actin | CACCACACCTTCTACAAC | TCTGGGTCATCTTCTCAC | 106 | 95.41 |
| IL-2 | CAACGGTGCACCTACTTCA | AGCTTGAGGTTCTCGGGATT | 115 | 99.93 |
| IL-4 | GTACCAGCCACTTCGTCCAT | GCTGCTGAGATTCCTGTCAA | 148 | 97.11 |
| IL-10 | CCTTGTCGGAAATGATCCAG | AGGGCAGAAAACGATGACAG | 150 | 98.68 |
| IL-17 | TTGTAAAGGCAGGGGTCATC | GGTGGAGCGCTTGTGATAAT | 149 | 103.91 |
| IFN-γ | GAACGGCAGCTCTGAGAAAC | GGTTAGATTTTGGCGACAGG | 131 | 98.02 |
| TGF-β1 | CATGAACCGGCCCTTCCT | GAAGTCAATGTAGAGCTGACGAACA | 126 | 98.98 |

**Table S2. Primer sequences for real-time PCR**

^*^ Amplification efficiency (%) = (10^-1/slope^ -1) ×100
