# Supplementary figures and images for: Hepatocellular carcinoma-associated antigen 59 of Haemonchus contortus modulates the functions of PBMCs and the differentiation and maturation of monocyte-derived dendritic cells of goats in vitro
Source: Parasit Vectors. 2019 Mar 14;12:105. doi: 10.1186/s13071-019-3375-1 (PMC6416944; doi:10.1186/s13071-019-3375-1)

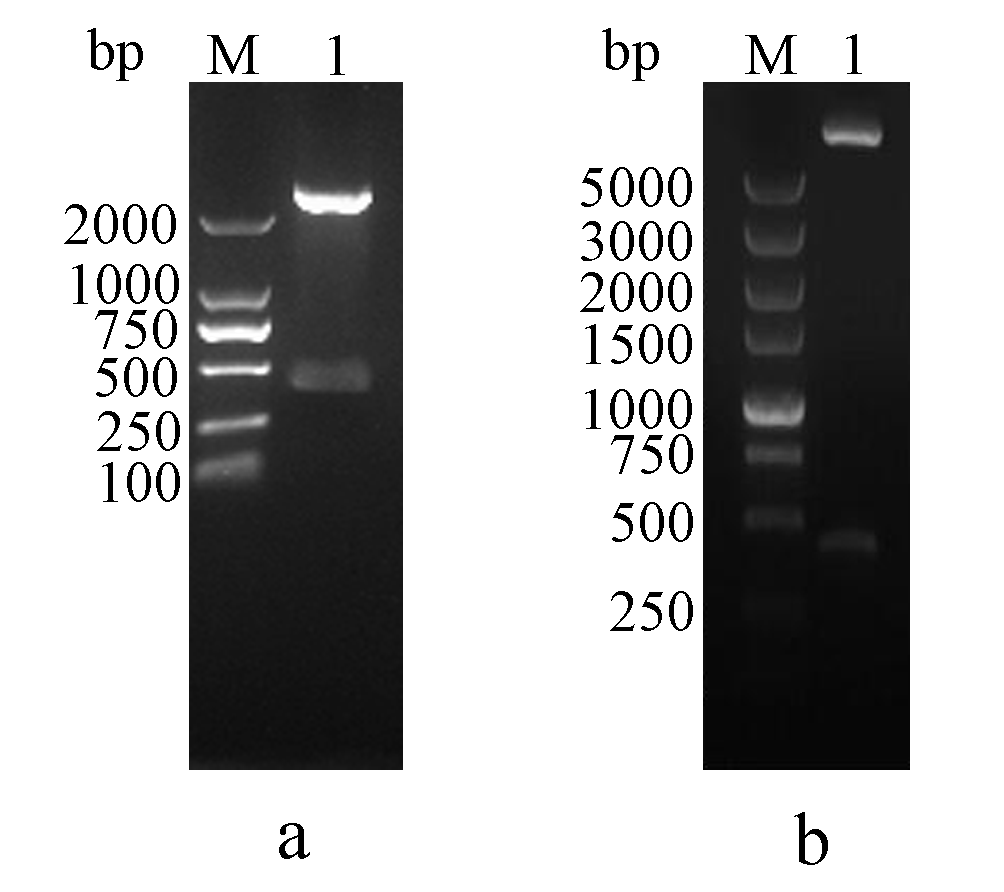

Supplement: Supplementary file 3 — Additional file 3: Figure S1. Double digestion analysis of HCA59. Lane M: DNA marker. a HCA59-19T plasmid digested by BamHI and HindIII. Lane 1: double digestion products with pMD19-T vector (2692 bp) and HCA59 (426 bp). b HCA59-pET32a plasmid digested by BamHI and HindIII. Lane 1: double digestion products with pET-32a vector (5900 bp) and HCA59 (426 bp). [file 13071_2019_3375_MOESM3_ESM.tif]

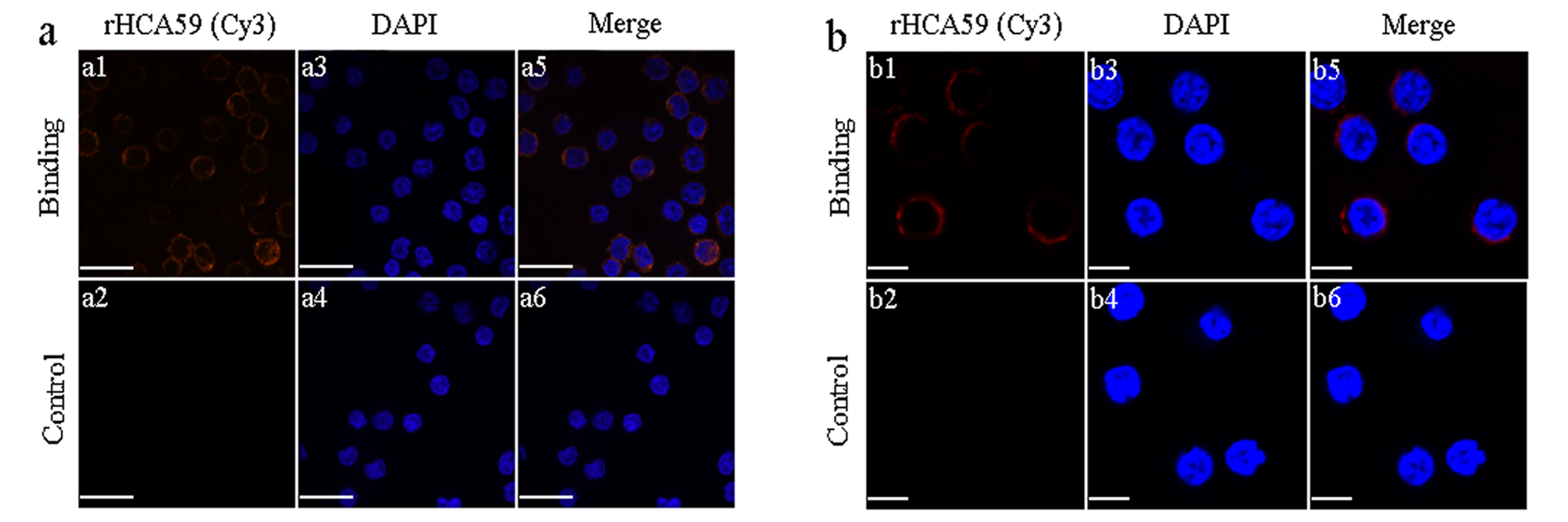

Supplement: Supplementary file 4 — Additional file 4: Figure S2. An immunofluorescence assay was conducted to determine if rHCA59 could bind to goat PBMCs (a) and md-DCs (b). Localization was performed by incubating DCs or PBMCs with rat anti-rHCA59 IgG or negative rat IgG (control). a1, a2, b1, b2 Staining of the target proteins (red) was visualized by Cy3-conjugated secondary antibody. a3, a4, b3, b4 Nuclei of the corresponding cells were visualized by DAPI (blue) staining. a5, a6, b5, b6 Merged, overlap of the red and blue channels. No red fluorescence was observed in the control group. Scale-bars: a, 10 μm; b, 5 μm. [file 13071_2019_3375_MOESM4_ESM.tif]

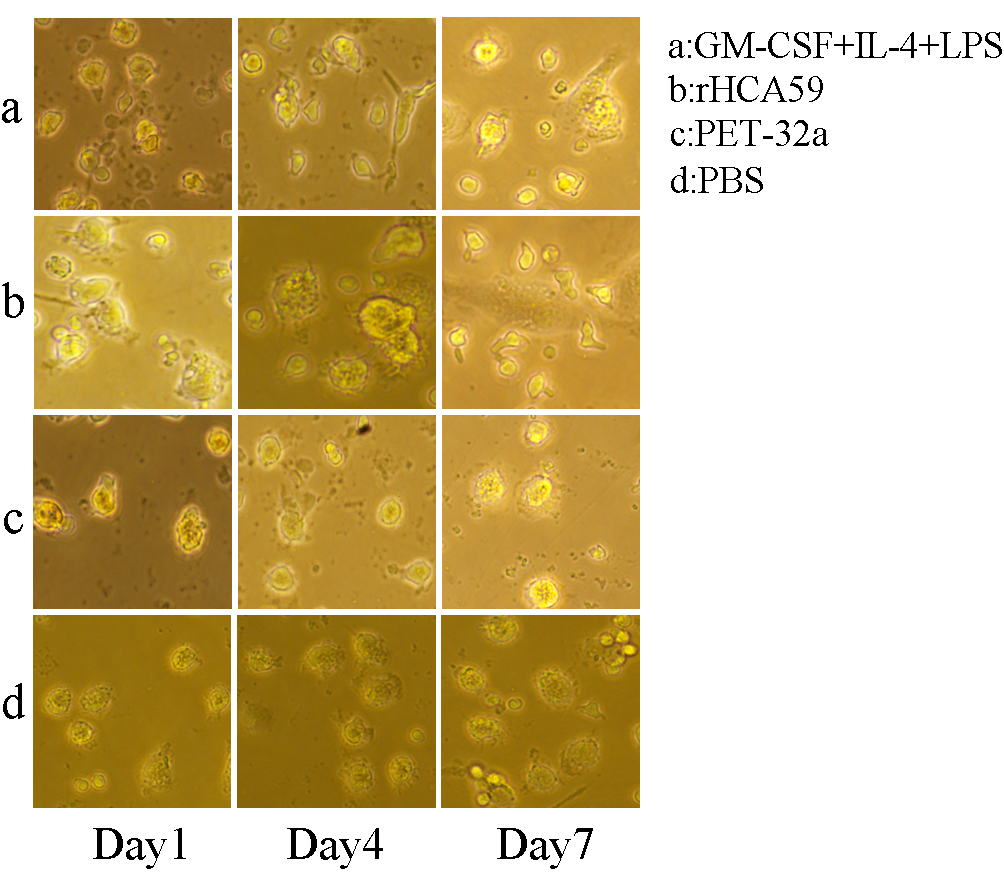

Supplement: Supplementary file 5 — Additional file 5: Figure S3. Generation of monocyte-derived dendritic cells from goat PBMCs. Morphology of cells was viewed by optical microscopy at 400× magnification. a Cells supplemented with GM-CSF+IL-4+LPS (positive group). b Cells treated with rHCA59 (tested group). c Cells treated with pET-32a protein (negative control). d Cells treated with PBS (blank control). [file 13071_2019_3375_MOESM5_ESM.tif]
